# Enhanced Pain Management With Combined EMLA Patch and Local Anesthetic Infiltration in Transradial Angiography

**DOI:** 10.1002/clc.70444

**Published:** 2026-08-24

**Authors:** Mohamad Amer Nashtar, Obayda Azizy, Jörn Trippe, Ali Canbay, Polykarpos Christos Patsalis, Martin Steinmetz

**Affiliations:** ^1^ Department of Internal Medicine, Division of Cardiology, Angiology and Internal Emergency Medicine, Knappschaft Kliniken University Hospital Bochum Ruhr University Bochum Bochum Germany; ^2^ Department of Internal Medicine, Division of Hepatology and Gastroenterology, Knappschaft Kliniken University Hospital Bochum Ruhr University Bochum Bochum Germany

**Keywords:** coronary angiography, EMLA patch, local anesthetic infiltration, patient comfort, radial artery access, topical anesthesia

## Abstract

**Background/Objectives:**

Pain at the radial access site remains common during coronary angiography. Evidence on topical anesthesia with EMLA patches is inconsistent, and the added value of combining EMLA with local lidocaine infiltration has not been fully clarified. This study compared the analgesic effectiveness of three strategies: EMLA patch, local lidocaine infiltration, and their combination.

**Methods:**

In this retrospective study, 275 patients undergoing elective transradial coronary angiography received either EMLA patch (*n* = 88), local infiltration (*n* = 96), or combined EMLA + infiltration (*n* = 91). Inclusion required the availability of complete data on pain assessments and access‐site complications. Pain intensity during the procedure and over 24 h was assessed using the Numeric Pain Rating Scale at five time points. Group comparisons used the Mann–Whitney *U*, Kruskal–Wallis, one‐way ANOVA, or chi‐square test. Linear regression was applied to identify predictors of pain.

**Results:**

Pain during angiography was lowest with combined anesthesia (median 0 [0–1]), compared with EMLA (1 [0–2]) or infiltration (3 [1–5]) (*p* < 0.001). Similar findings were observed for the 24 h cumulative pain score. An EMLA application time of 100–300 min was associated with substantially lower pain than shorter or longer durations (both *p* < 0.001). Obesity (BMI ≥ 30 kg/m^2^) independently predicted higher procedural pain (B = 0.96; *p* = 0.020) and higher 24 h cumulative pain (B = 2.70; *p* = 0.002).

**Conclusions:**

Combined EMLA patch plus local lidocaine infiltration was associated with superior analgesia during and after transradial coronary angiography compared with either method alone. An EMLA application time of 100–300 min may optimize analgesic effectiveness, while patients with obesity may benefit from tailored pain‐management strategies.

## Practitioner points

- Combining an EMLA patch with local lidocaine infiltration may provide the greatest reduction in access‐site pain during and after elective transradial coronary angiography.
- Adequate pre‐procedural EMLA application time appears important; in this cohort, application for 100–300 min was associated with the lowest pain scores.
- Patients with obesity may experience higher access‐site pain and may benefit from individualized local anesthesia strategies.

## Graphical abstract

Among 275 elective transradial coronary angiography procedures, combined EMLA patch plus local lidocaine infiltration was associated with the lowest procedural and 24 h access‐site pain. EMLA application for 100–300 min was associated with lower pain, while BMI ≥ 30 kg/m^2^ predicted higher pain, supporting individualized analgesic strategies.

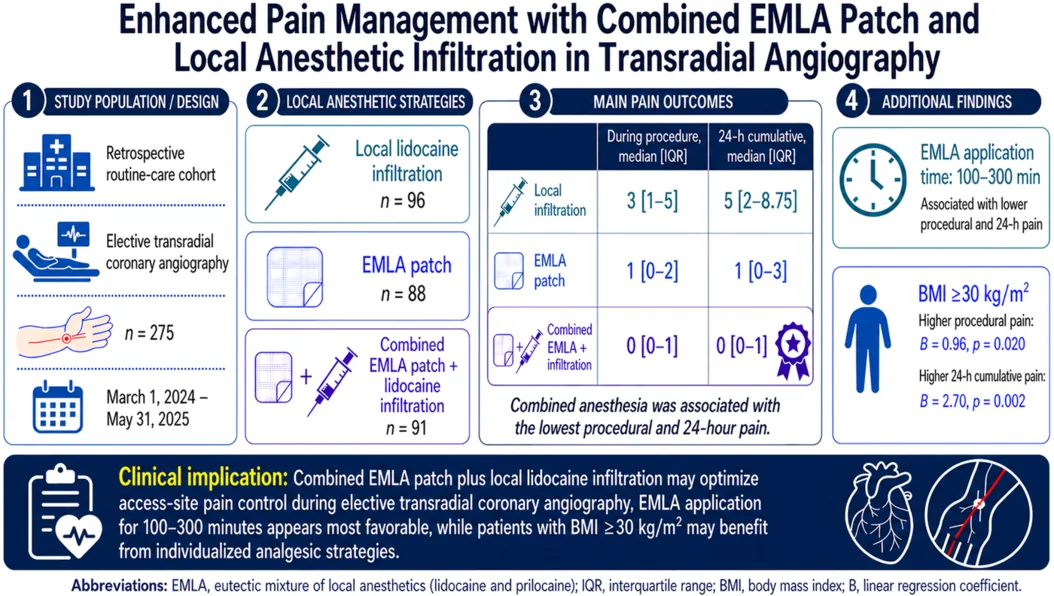

**Abbreviations**
B: regression coefficient (linear regression)
BMI: body mass index
CI: confidence interval
EMLA: eutectic mixture of local anesthetics (lidocaine/prilocaine)
kg/m^2^
: kilograms per square meter (BMI unit)
min: minutes
NPRS: numeric pain rating scale
PCI: percutaneous coronary intervention
r: Spearman's rank correlation coefficient
SD: standard deviation

## 1 Introduction

Radial artery access has become the standard approach for coronary angiography in many centers worldwide due to its lower complication rates, reduced bleeding risk, and earlier patient mobilization and discharge compared with femoral access [1, 2, 3]. Despite these advantages, patients may still experience discomfort and pain during radial artery cannulation, underscoring the need for effective pain‐management strategies [4].

Local infiltration with lidocaine is commonly used to reduce pain at the access site. However, the infiltration itself can cause discomfort and anxiety [5]. Topical anesthetics such as lidocaine/prilocaine eutectic cream (EMLA) offer a noninvasive alternative that may reduce both needle‐related pain and general discomfort during and after the procedure [5, 6]. Nevertheless, evidence regarding the analgesic benefit of topical anesthetic agents in arterial puncture remains inconsistent, with some studies reporting no significant reduction in puncture‐related pain with EMLA or amethocaine gel [7, 8]. Therefore, the optimal role of topical anesthesia, particularly in combination with local infiltration, remains insufficiently defined.

This retrospective study aimed to evaluate and compare the analgesic effectiveness of three local anesthetic strategies—EMLA patch, local lidocaine infiltration, and their combination—for pain management in patients undergoing elective transradial coronary angiography, and to investigate potential predictors of pain.

## 2 Methods

### 2.1 Study Design and Patient Selection

This study was designed as a retrospective analysis of prospectively collected routine‐care data from a tertiary care center (Knappschaft Kliniken, University Hospital Bochum, Germany). A total of 275 patient records from individuals who underwent elective coronary angiography via radial artery access at our center between March 1, 2024, and May 31, 2025, were retrieved and analyzed. Eligible patients were adults undergoing elective transradial coronary angiography with complete documentation of the local anesthetic strategy, standardized pain assessment, and local access‐site side effects. Exclusion criteria were age < 18 years, emergency coronary angiography, non‐radial access, incomplete or missing pain assessment data, unclear allocation to one of the predefined anesthetic strategies, or missing documentation of local access‐site side effects.

All procedures were performed via proximal radial access by experienced interventional cardiologists. All patients received intra‐arterial verapamil (2.5 mg) and nitroglycerin (200 µg) as standard vasodilators/spasmolytic agents. The patient population included in the analysis underwent one of the following local anesthetic strategies: local subcutaneous infiltration with lidocaine (2.5 mL of 1% lidocaine [25 mg], *n* = 96), topical application of an EMLA patch (5% eutectic mixture containing lidocaine 25 mg and prilocaine 25 mg per 10 cm^2^ patch, *n* = 88), or a combination of both methods (*n* = 91). The choice of local anesthetic strategy was not randomized but followed routine clinical practice and operator preference, influenced by scheduling logistics and perceived patient needs.

At our center, pain perception and local side effects are systematically collected as part of routine clinical care and quality management during elective coronary angiography using a structured questionnaire, which is provided as File S1. Although the questionnaire itself has not been externally validated as an independent instrument, pain intensity was assessed using the established 11‐point Numeric Pain Rating Scale (NPRS), ranging from 0 (“no pain”) to 10 (“worst imaginable pain”). The NPRS is widely used for unidimensional self‐reported pain‐intensity assessment and has demonstrated favorable validity, reliability, feasibility, and responsiveness in adult populations and clinical pain research [9, 10]. Patients rated access‐site pain during the procedure and at 1, 6, 12, and 24 h after completion of angiography. The summation of the NPRS scores from all five time points was used to calculate a 24 h cumulative pain score, ranging from 0 to 50.

Demographic, clinical, and procedural data were extracted from the hospital information system and procedural documentation. These included age, sex, body mass index (BMI), duration of catheterization, performance of an intervention, and relevant comorbidities, including diabetes mellitus, hypertension, smoking status, hyperlipidemia, chronic kidney disease, coronary artery disease, and peripheral arterial disease.

The number of puncture attempts, use of ultrasound guidance, and radial artery diameter were not consistently documented in a structured format and, therefore, were not available for inclusion in the present analysis.

No formal a priori sample size calculation was performed because the study was based on a retrospective analysis of available routine‐care data. Instead, all eligible patients during the study period who fulfilled the predefined inclusion criteria and had complete standardized pain assessments were included. Thus, the final sample size was determined by data availability and comprised 275 patients with broadly balanced group sizes across the three anesthetic strategies. Accordingly, the analyses should be interpreted as exploratory and hypothesis‐generating.

### 2.2 Statistical Methods

All data were transferred from the hospital information system to Excel. Statistical analyses were performed using GraphPad Prism (version 10.5, GraphPad Software, San Diego, CA, USA). Continuous variables with a normal distribution were expressed as the mean ± standard deviation (SD), while non‐normally distributed variables were summarized as the median (Q1–Q3). Categorical variables were expressed as absolute numbers (*n*) and percentages (%). For continuous variables, two‐group comparisons were conducted using the Mann–Whitney *U* test. Comparisons across three groups were performed using the Kruskal–Wallis test or one‐way ANOVA, as appropriate. Categorical variables were analyzed using the chi‐square test. To assess the correlations, Spearman's rank correlation coefficient was utilized. Linear regression analysis was conducted to determine potential predictors of increased pain intensity and limited analgesic efficacy of the local anesthetic strategies. A two‐sided *p* value less than 0.05 was considered statistically significant.

## 3 Results

### 3.1 Characteristics of the Investigated Patients

Baseline demographic and clinical characteristics were broadly similar across the three anesthetic strategy groups, with no statistically significant differences in age, sex, cardiovascular risk factors, coronary artery disease, or procedural intervention rates (all *p* > 0.05).

The mean age of the study population was 72.1 ± 12.2 years. Overall, 174 patients (63.3%) were male, and 173 patients (62.9%) had a BMI ≥ 25 kg/m^2^. Percutaneous coronary intervention (PCI), including stent implantation or balloon dilatation, was performed in 183 patients (66.5%), whereas 92 patients (33.5%) underwent diagnostic coronary angiography only. The mean duration of catheterization was 42 ± 15.5 min.

A detailed overview of baseline characteristics and statistical comparisons is presented in Table 1.

**Table 1:** Baseline characteristics of the study cohort stratified by local anesthetic strategy.

Variable	EMLA patch (*n* = 88)	Local infiltration (*n* = 96)	EMLA patch + local infiltration (*n* = 91)	Total (*n* = 275)	*p* value
Age, years	74.2 ± 10.5	71.8 ± 12.7	70.3 ± 12.9	72.1 ± 12.2	0.0905
Male sex	54 (61.4)	60 (62.5)	60 (65.9)	174 (63.3)	0.8025
BMI ≥ 30, kg/m^2^	14 (15.9)	12 (12.5)	15 (16.5)	41 (14.9)	0.7095
BMI 25–29.9, kg/m^2^	44 (50.0)	49 (51.0)	39 (42.9)	132 (48.0)	0.4816
BMI < 25, kg/m^2^	30 (34.1)	35 (36.5)	37 (40.7)	102 (37.1)	0.653
Duration of catheterization, min	40 ± 16	42 ± 13	44 ± 17	42 ± 15.5	0.132
Intervention	56 (63.6)	64 (66.7)	63 (69.2)	183 (66.5)	0.7298
Diabetes	28 (31.8)	29 (30.2)	28 (30.8)	85 (30.9)	0.9719
Hypertension	58 (65.9)	57 (59.4)	64 (70.3)	179 (65.1)	0.2857
Smoking	31 (35.2)	28 (29.2)	20 (22.0)	79 (28.7)	0.1459
Hyperlipidemia	45 (51.1)	54 (56.2)	45 (49.5)	144 (52.4)	0.6238
Chronic kidney disease	15 (17.0)	13 (13.5)	19 (20.9)	47 (17.1)	0.4116
Coronary artery disease	77 (87.5)	86 (89.6)	76 (83.5)	239 (86.9)	0.4605
Peripheral arterial disease	5 (5.7)	8 (8.3)	7 (7.7)	20 (7.3)	0.7733

*Note:* This table summarizes demographic and clinical characteristics of patients undergoing coronary catheterization, stratified by anesthetic strategy: EMLA patch, local infiltration, and the combination of both. Continuous variables are presented as mean ± standard deviation; categorical variables as *n* (%). *p* values were calculated using one‐way ANOVA for continuous variables and the chi‐square test for categorical variables. The variable “Intervention” refers to patients who underwent balloon dilatation or stent implantation during the procedure; all remaining patients received diagnostic coronary angiography only.

Abbreviations: BMI, body mass index; min, minutes.

### 3.2 Subjective Perception of Pain During and After Transradial Coronary Angiography

**Figure 1:**
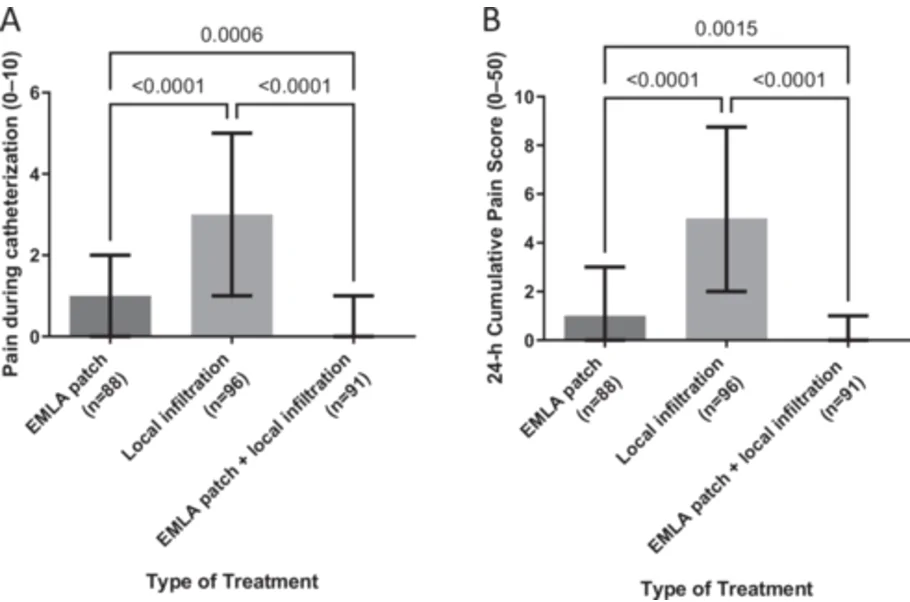
Pain intensity during and after catheterization across anesthetic strategies. Median pain intensity with interquartile ranges during catheterization (A) and over the following 24 h (B), shown across the three local anesthetic strategies: EMLA patch (*n* = 88), local infiltration (*n* = 96), and the combination of both (*n* = 91). *p* values were derived from the Kruskal–Wallis test.

The median pain intensity during the procedure was 3 (1–5) in the local infiltration group, 1 (0–2) in the EMLA group, and lowest in the combination group, which received both EMLA and local infiltration, with a median of 0 (0–1). Pain intensity in the combination group was significantly lower compared to local infiltration alone (*p* < 0.0001) and EMLA alone (*p* = 0.0006). Additionally, the EMLA group reported significantly lower pain levels than the local infiltration group (*p* < 0.0001) (Figure 1A).

A similar trend was observed in the analysis of the 24 h cumulative pain score, with median values of 5 (2–8.75) in the local infiltration group, 1 (0–3) in the EMLA group, and 0 (0–1) in the combination group. The combination group showed significantly lower cumulative pain scores compared to both the local infiltration group (*p* < 0.0001) and the EMLA group (*p* = 0.0015), while the EMLA group also demonstrated significantly lower scores than the local infiltration group (*p* < 0.0001) (Figure 1B).

### 3.3 Pre‐Procedural Contact Time of EMLA and Anesthetic Effectiveness

**Figure 2:**
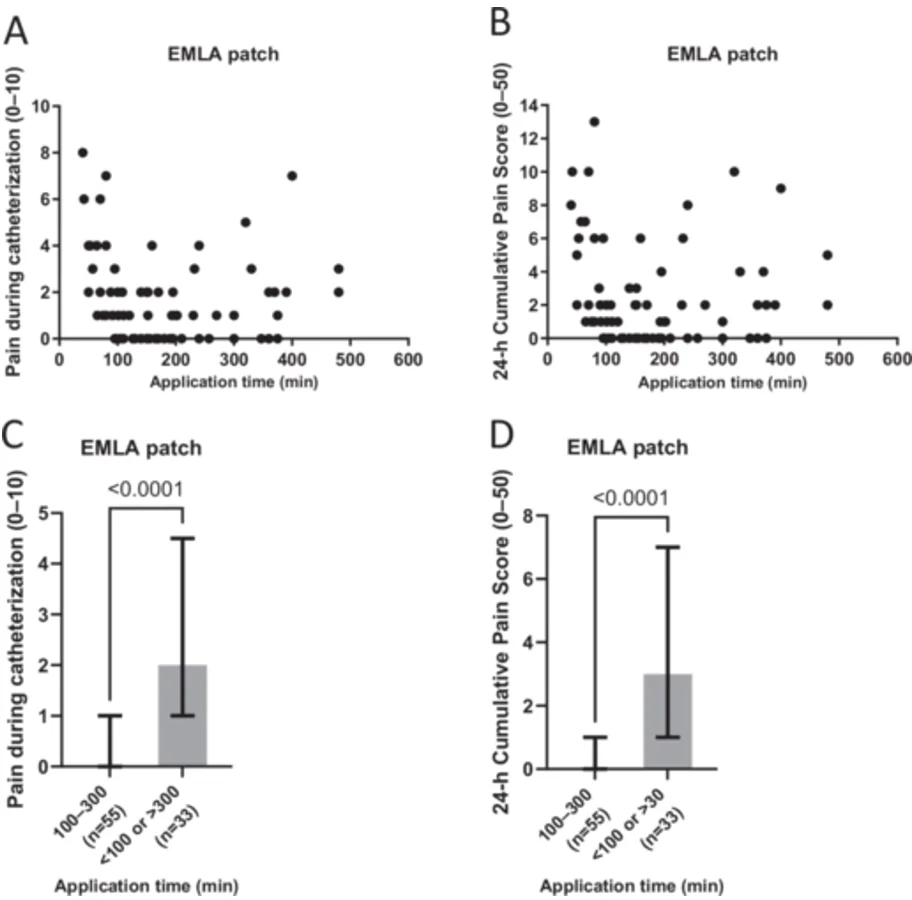
Pain intensity in relation to EMLA patch application time during and after catheterization. Scatterplots showing the distribution of pain scores in relation to the application time of the EMLA patch (*n* = 88) during catheterization (A) and over the following 24 h (B). Median pain intensity with interquartile ranges during catheterization (C) and over the following 24 h (D), stratified by EMLA patch application time: 100–300 min (*n* = 55) versus < 100 or > 300 min (*n* = 33). *p* values were derived from the Mann–Whitney *U* test.

To explore the association between pre‐procedural EMLA application time and anesthetic effectiveness, we first analyzed patients receiving EMLA patches alone. A significant but weak inverse correlation was observed between the duration of EMLA patch application and pain intensity during the procedure (Spearman's *r* = –0.239; 95% CI: –0.432 to –0.024; *p* = 0.025). Similarly, a weak inverse correlation was identified between the duration of EMLA application and the 24 h cumulative pain score (Spearman's *r* = –0.213; 95% CI: –0.410 to 0.003; *p* = 0.046) (Figure 2A,B). The lowest recorded pain intensity was observed when the application duration ranged between 100 and 300 min. Specifically, the median pain intensity during the procedure in this subgroup was 0 (0–1), which was significantly lower than in patients with application durations outside this range (< 100 or > 300 min), who reported a median of 2 (1–4.5) (*p* < 0.0001). A similar pattern was observed for the 24 h cumulative pain score, with a median of 0 (0–1) for the 100–300 min group, compared to 3 (1–7) for those outside this range (*p* < 0.0001) (Figure 2C,D).

**Figure 3:**
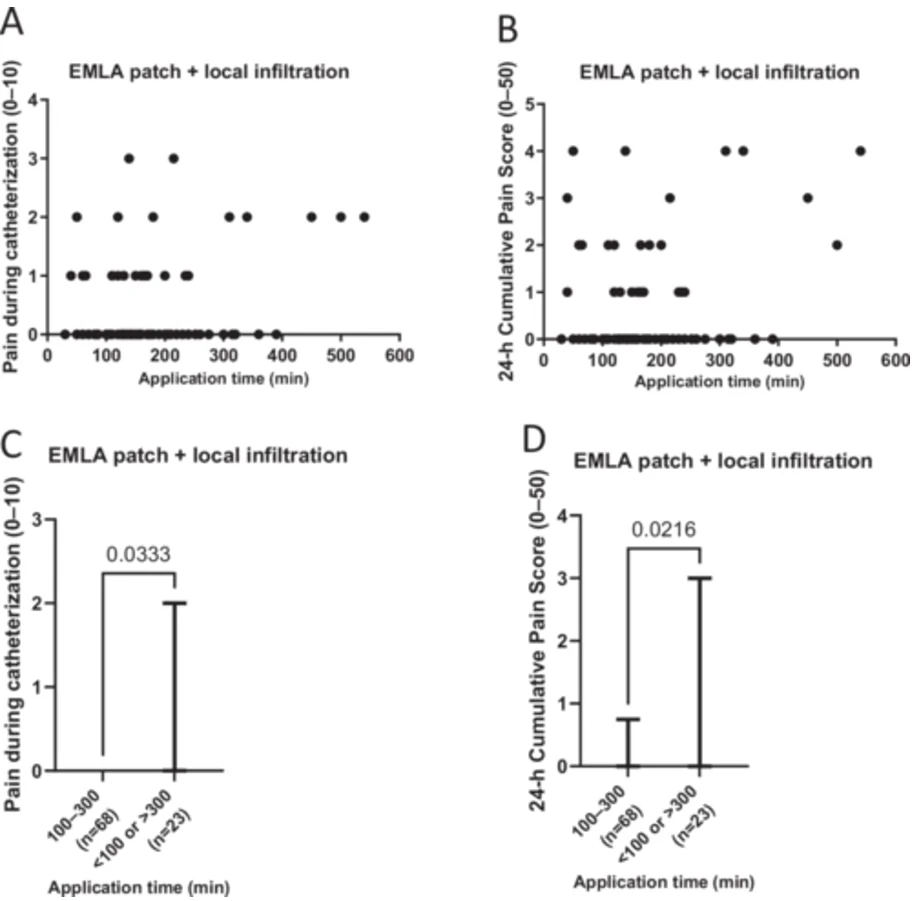
Pain intensity in relation to EMLA patch application time in the combination anesthesia group. Scatterplots showing the distribution of pain scores in relation to EMLA patch application time in the combination group (EMLA patch + local infiltration, *n* = 91) during catheterization (A) and over the following 24 h (B). Median pain intensity with interquartile ranges in the combination group during catheterization (C) and over the following 24 h (D), stratified by EMLA patch application time: 100–300 min (*n* = 68) versus < 100 or > 300 min (*n* = 23). *p* values were derived from the Mann–Whitney *U* test.

In the EMLA plus local infiltration group, no significant correlation was identified between the duration of EMLA patch application and pain intensity during the procedure (Spearman's *r* = 0.093; 95% CI: –0.121 to 0.299; *p* = 0.379), nor with the 24 h cumulative pain score (Spearman's *r* = 0.094; 95% CI: –0.121 to 0.300; *p* = 0.377) (Figure 3A,B).

A comparative analysis within the combination group was conducted between patients with an EMLA application duration between 100 and 300 min and those with shorter (< 100 min) or longer (> 300 min) durations. Median pain intensity during coronary angiography was significantly lower in the 100–300 min group than in patients with shorter or longer application durations (0 [0–0] vs. 0 [0–2]; *p* = 0.0333). Similarly, the 24 h cumulative pain score was significantly lower in this group (0 [0–0.75] vs. 0 [0–3]; *p* = 0.0216), consistent with the trend observed in the EMLA‐only group (Figure 3C,D).

### 3.4 Potential Influential Factors and Confounders

The findings revealed that neither age, sex, duration of catheterization, cardiovascular risk factors—including diabetes, hypertension, smoking, hyperlipidemia, and chronic kidney disease—nor the presence of atherosclerotic cardiovascular disease, or the performance of an intervention (PCI vs. no PCI), showed a significant association with pain perception, either during the procedure or over the following 24 h, according to both correlation and regression analyses (all *p* > 0.1). A BMI ≥ 30 kg/m^2^ demonstrated a significant and positive association with pain intensity during coronary angiography (Spearman's *r* = 0.142, 95% CI: 0.020–0.259; *p* = 0.0186), with a corresponding linear regression coefficient of B = 0.962 (95% CI: 0.152–1.772; *p* = 0.0201). A similar association was observed for the cumulative 24 h pain score (Spearman's *r* = 0.142, 95% CI: 0.020–0.259; *p* = 0.0188; B = 2.700, 95% CI: 1.018–4.383; *p* = 0.0018). After adjustment for age, sex, duration of catheterization, and procedural intervention status, BMI ≥ 30 kg/m^2^ remained independently associated with higher pain intensity during the procedure and with increased 24 h cumulative pain scores, with effect estimates comparable to the unadjusted analyses (data not shown).

A comparative analysis was conducted between patients with a BMI ≥ 30 kg/m^2^ and those with a BMI < 30 kg/m^2^. The analysis revealed that the higher‐BMI group exhibited significantly higher pain scores in both the EMLA and EMLA plus local infiltration groups. In the local infiltration group, pain scores also tended to be higher among patients with BMI ≥ 30 kg/m^2^, although these differences did not reach statistical significance.

**Figure 4:**
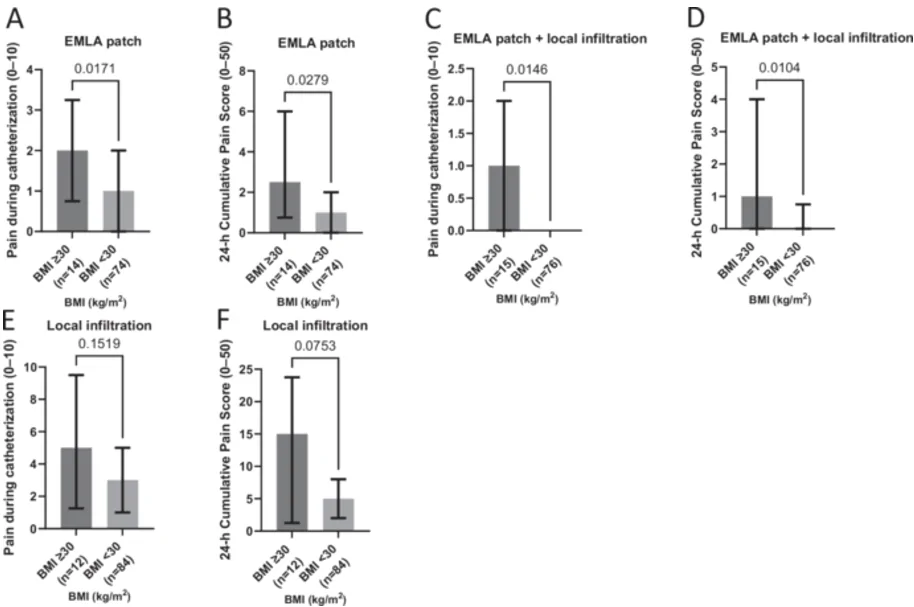
Pain intensity by BMI category across anesthetic strategies during and after catheterization. Median pain intensity with interquartile ranges during catheterization (A, C, E) and over the following 24 h (B, D, F), stratified by body mass index (BMI ≥ 30 vs. BMI < 30), across the three local anesthetic strategies: EMLA patch (BMI ≥ 30: *n* = 14; BMI < 30: *n* = 74), local infiltration (BMI ≥ 30: *n* = 12; BMI < 30: *n* = 84), and combined application (BMI ≥ 30: *n* = 15; BMI < 30: *n* = 76). *p* values were derived from the Mann–Whitney *U* test.

In the EMLA group, patients with a BMI ≥ 30 kg/m^2^ reported higher pain scores during the procedure (median 2 [0.75–3.25] vs. 1 [0–2]; *p* = 0.0171) and higher 24 h cumulative pain scores (2.5 [0.75–6] vs. 1 [0–2]; *p* = 0.0279) (Figure 4A,B). In the EMLA plus local infiltration group, analogous findings were observed. The higher BMI group exhibited significantly higher pain during the procedure (1 [0–2] vs. 0 [0–0]; *p* = 0.0146) and higher 24 h cumulative pain scores (1 [0–4] vs. 0 [0–0.75]; *p* = 0.0104) (Figure 4C,D). In the local infiltration group, patients with a BMI ≥ 30 kg/m^2^ exhibited higher median scores during the procedure (5 [1.25–9.5] vs. 3 [1–5]; *p* = 0.1519) and over 24 h (15 [1.25–23.75] vs. 5 [2–8]; *p* = 0.0753). However, these differences did not reach statistical significance (Figure 4E,F).

Across all three local anesthetic strategies, no clinically relevant adverse effects were observed. Notably, there were no cases of allergic reactions, prolonged numbness, local infections, or access‐site complications attributable to the anesthetic method.

## 4 Discussion

The present study investigated the effectiveness of various analgesic strategies in reducing pain during and after coronary angiography with radial access. The analgesic strategies included a topical EMLA patch, local anesthetic infiltration, and their combination. The main finding was that combined EMLA patch plus local lidocaine infiltration was associated with the lowest pain scores during the procedure and over the subsequent 24 h. The findings suggested lower pain scores with EMLA compared with local infiltration, indicating that preprocedural topical anesthesia may enhance patient comfort. Furthermore, a higher BMI (≥ 30 kg/m^2^) was found to be significantly associated with increased pain intensity, particularly in patients receiving EMLA or combination therapy.

A body of research has demonstrated that the administration of adequate local anesthesia is associated with a reduction in patient discomfort and anxiety regarding the procedure. This phenomenon has the potential to influence success rates and treatment cooperation [6, 11, 12]. However, the utilization of topical anesthetics, particularly EMLA, has been predominantly confined to pediatric, dermatological, or minor surgical contexts.

A body of literature has emerged regarding the efficacy of EMLA in reducing pain during radial approach procedures. However, the results of these studies have been inconsistent, suggesting that further investigation is necessary to determine the effectiveness of EMLA in this context. A number of studies reported comparable analgesic effects between EMLA and local infiltration [13], while others have demonstrated superior pain reduction with EMLA [6, 14]. Conversely, subsequent research has revealed no substantial benefits of EMLA in alleviating procedural pain [7]. Smith et al. found that the combination of EMLA and local infiltration did not result in enhanced pain reduction compared to EMLA alone [14]. In contrast, our findings demonstrate that the combination of EMLA and lidocaine infiltration was the most effective strategy, achieving superior pain relief compared to either EMLA or local infiltration alone. The observed superiority in analgesia of the combination strategy is likely attributable to the effects of both surface and deeper tissue anesthesia. This is a salient consideration in radial access, wherein the skin, subcutaneous tissue, and arterial wall are all traversed. The inverse correlation between EMLA application time and pain scores, though weak, suggests that prolonging exposure may enhance its analgesic effect. These findings support the importance of adequate application time (typically ≥ 60 min) for achieving optimal efficacy. The analysis revealed that the application of EMLA for a duration between 100 and 300 min yielded the most efficacious pain mitigation, both within the EMLA‐only group and in the combination group. These findings are consistent with those of Russell et al., who conducted a comparative analysis of the analgesic efficacy of EMLA with application durations of 60 min versus at least 90 min, reporting superior pain control with longer application times [5].

The positive correlation between obesity (BMI ≥ 30 kg/m^2^) and pain intensity may be attributable to factors such as augmented subcutaneous tissue thickness, modified pain thresholds, and technical challenges in vascular access. The relationship between obesity, body fat distribution, and pain perception remains incompletely understood, with prior studies reporting divergent findings. Some studies have suggested a positive correlation between increased subcutaneous fat and reduced pain perception, possibly due to a cushioning effect [15]. However, other studies have reported heightened pain sensitivity in individuals with obesity [16, 17]. Furthermore, some investigations have found no significant correlation between BMI and pain intensity [18]. Notably, variables such as age, sex, duration of catheterization, cardiovascular risk factors, the presence of atherosclerotic disease, and intervention status (PCI vs. diagnostic angiography) were not significantly associated with pain perception. These findings suggest that effective pain management should be guided more by factors such as BMI and access site preparation, as common comorbidities and procedural complexity did not significantly influence pain perception.

A significant strength of this study is the real‐world nature of the cohort and the standardized pain assessment at multiple time points, including the novel 24 h cumulative pain score. However, several limitations must be acknowledged. First, the retrospective design inherently restricts control of confounding variables and limits causal inference. Second, the absence of randomization may introduce selection bias. Third, patient‐reported pain is inherently subjective and can be influenced by anxiety, prior procedural experiences, or cultural factors. Fourth, although pain intensity was assessed using the established 11‐point NPRS, the structured questionnaire used for routine clinical care and quality management was not formally validated as an independent instrument.

Taken together, these findings highlight the clinical relevance of optimizing local anesthesia protocols. Nevertheless, further evaluation of cost‐effectiveness, procedural workflow, and patient satisfaction is needed before broader implementation can be recommended.

## 5 Conclusions

In the context of increasing utilization of radial access for coronary angiography, minimizing discomfort at the access site is essential to ensure optimal patient care. The findings of this study suggest that EMLA patches, particularly in conjunction with lidocaine infiltration, are associated with lower access‐site pain compared with either method alone. Patients with obesity may experience higher pain levels and could benefit from tailored local anesthesia strategies. Future prospective randomized studies are warranted to confirm these findings and define optimal implementation in routine clinical practice.

## Author Contributions

**Mohamad Amer Nashtar:** data curation, formal analysis, investigation, methodology, writing – original draft. **Obayda Azizy:** writing – review and editing. **Jörn Trippe:** writing – review and editing. **Ali Canbay:** writing – review and editing. **Polykarpos Christos Patsalis:** writing – review and editing. **Martin Steinmetz:** conceptualization, investigation, data curation, supervision, resources, writing – review and editing.

## Funding

The authors have nothing to report.

## Ethics Statement

This study was performed in accordance with the ethical principles and standards of the 1964 Declaration of Helsinki and the guidelines of the International Council for Harmonisation Good Clinical Practice. The study was approved by the Ethics Committee of the Faculty of Medicine at Ruhr University Bochum (approval number: 460857‐BR; approval date: August 21, 2018). Because the analysis was based on anonymized routine‐care data that do not allow the identification of individuals under Article 4(1) and Recital 26 of the EU General Data Protection Regulation (GDPR), no separate informed consent was required. This interpretation is consistent with the ethical review standards of the local ethics committee and applicable German data‐protection regulations.

## Consent

The authors have nothing to report.

## Conflicts of Interest

The authors declare no conflicts of interest.

## Supporting information


Supporting File

## Data Availability

Data supporting the findings of this study can be obtained from the corresponding author upon reasonable request and in accordance with institutional and ethical regulations.
