# Supplementary material for: Enhanced Pain Management With Combined EMLA Patch and Local Anesthetic Infiltration in Transradial Angiography
Source: Clin Cardiol. 2026 Aug 24;49(8):e70444. doi: 10.1002/clc.70444 (PMC13501893; doi:10.1002/clc.70444)
Supplement: Supplementary file 1 [file CLC-49-e70444-s001.docx]

**Questionnaire for Pain Assessment After Transradial Coronary Angiography**

*Routine clinical care and quality-management questionnaire*

**Note.** This structured questionnaire was used as part of routine clinical care and quality management after elective transradial coronary angiography. Pain intensity was assessed using the 11-point Numeric Pain Rating Scale (NPRS), ranging from 0 ("no pain") to 10 ("worst imaginable pain").

**General information**

| Patient/case ID: |  | Age, years: |  |
| --- | --- | --- | --- |
| Sex: | ☐ Male ☐ Female ☐ Diverse / other | Date of coronary angiography: |  |

**Pain assessment at the radial access site**

Do you currently have pain at the radial access site? ☐ Yes	☐ No

**Please rate the intensity of pain at the radial access site at the following time points using the 0-10 NPRS:** 0 = no pain; 10 = worst imaginable pain.

| **Time point** | **0** | **1** | **2** | **3** | **4** | **5** | **6** | **7** | **8** | **9** | **10** |
| --- | --- | --- | --- | --- | --- | --- | --- | --- | --- | --- | --- |
| During coronary angiography | ☐ | ☐ | ☐ | ☐ | ☐ | ☐ | ☐ | ☐ | ☐ | ☐ | ☐ |
| 1 hour after coronary angiography | ☐ | ☐ | ☐ | ☐ | ☐ | ☐ | ☐ | ☐ | ☐ | ☐ | ☐ |
| 6 hours after coronary angiography | ☐ | ☐ | ☐ | ☐ | ☐ | ☐ | ☐ | ☐ | ☐ | ☐ | ☐ |
| 12 hours after coronary angiography | ☐ | ☐ | ☐ | ☐ | ☐ | ☐ | ☐ | ☐ | ☐ | ☐ | ☐ |
| 24 hours after coronary angiography | ☐ | ☐ | ☐ | ☐ | ☐ | ☐ | ☐ | ☐ | ☐ | ☐ | ☐ |

**Pain characteristics**

How would you describe the pain at the radial access site? Multiple answers are possible.

| ☐ Sharp/stabbing | ☐ Burning | ☐ Dull | ☐ Pulling/tight |
| --- | --- | --- | --- |
| ☐ Throbbing | ☐ No pain | ☐ Other: ______________________________ |  |

**Local side effects or adverse events**

Did you notice any local side effects or adverse events at the radial access site or after local anesthetic application, such as skin irritation, redness, itching, swelling, prolonged numbness, or other symptoms?

☐ Yes	☐ No	If yes, please describe: __________________________________________________________________________________________

________________________________________________________________________________________________________________________

*Thank you for completing this questionnaire.*
